# The relationship between physical activity and appetite among older adults — A scoping review

**DOI:** 10.1016/j.jnha.2025.100538

**Published:** 2025-03-23

**Authors:** Annelie Turesson, Philipe De Souto Barreto, Margaretha Nydahl, Afsaneh Koochek

**Affiliations:** a Department of Food Studies, Nutrition and Dietetics, Uppsala University, Uppsala, Sweden; b IHU HealthAge, Toulouse, France; c CERPOP UMR 1295, University of Toulouse, INSERM, UPS, Toulouse, France; d Institute on Aging of the Toulouse University Hospital (CHU Toulouse), Toulouse, France

**Keywords:** Anorexia of aging, Hunger, Satiety, Older people, Exercise

## Abstract

**Background:**

Appetite loss among older adults is common and may lead to malnutrition, reduced function and frailty. Regular physical activity may help regulate appetite and enhance overall wellbeing. However, evidence concerning the relationship between physical activity and appetite in older adults remains scarce.

**Objectives:**

This study aims to examine the existing literature for the relationship between appetite and physical activity among older adults, contributing to the identification of existing research gaps.

**Method:**

A scoping review was conducted using a block search strategy with keywords including physical activity, exercise, appetite, and older adults. Searches were performed in Web of Science, PubMed, and CINAHL on November 23, 2023. The inclusion criteria were: English language, human studies, population aged 55+ without severe disease, addressing both appetite and physical activity, original articles, and not qualitative studies.

**Results:**

Of 1190 articles identified, 25 were included. Females comprised 52% of the participants and most studies focused on community-dwelling older adults, primarily in the US and northern Europe. Research design and methodologies varied widely. Of the 25 studies, 14 were cross-sectional studies, seven randomized controlled trials, three non-randomized controlled trials, and one longitudinal observational study. Associations between physical activity and appetite were found in a longitudinal observational study including 186 participants, two randomized trials involving higher-intensity exercise, all non-randomized studies, and 10 cross-sectional studies.

**Conclusion:**

Although an association between appetite and physical activity among older adults has been observed, the lack of randomized controlled trials limits conclusions regarding whether exercise can effectively regulate appetite in older adults.

## 1 Introduction

Age-related decline in appetite and food intake, commonly referred to as the anorexia of aging [1], is prevalent among older adults, affecting approximately 10% of community-dwelling older adults and over 30% of nursing home residents. Appetite loss is linked to negative health outcomes, such as depression, reduced energy intake, weight loss, frailty and an increased risk of mortality [[2], [3], [4], [5]]. This, together with high prevalence, highlights the critical role poor appetite may have in the health of the older population.

Research indicates a link between exercise and improved appetite. For example, studies suggest that exercise can speed up gastric emptying and increase the levels of ghrelin, an appetite-regulating hormone that signals hunger [[6], [7], [8]]. However, most of the literature focuses on younger adults, highlighting a gap in understanding the association between physical activity and appetite in older adults. Inflammatory markers and medication use are more prevalent in older adults, which both may negatively impact appetite. Additionally, factors such as reduced saliva production, sensory changes, and delayed gastric emptying are common in this population, which may also affect appetite [9,10]. Evidence regarding the interplay between physical activity and appetite in older adults remains limited and unclear.

Despite existing reviews on related topics, no review has examined the direct link between physical activity and appetite in older adults. The few existing systematic reviews [11,12] focus on appetite and energy intake, making it difficult to isolate the association between appetite and physical activity itself. Furthermore, previous reviews have largely focused on younger populations [13]. This leaves a gap in understanding how age-related factors, like inflammation, medication use, and sensory decline [14,15], interact with physical activity to influence appetite. Additionally, recent research has not been fully addressed in existing reviews, underscoring the need for updates. Given the high prevalence and serious consequences of appetite loss in this population, updated research on this is essential. This scoping review aims to examine the existing literature concerning appetite and physical activity among older adults. This review also aims to identify existing research gaps and offer potential directions for future investigations within this area.

## 2 Method

This scoping review was pre-registered on the International Platform of Registered Systematic Review and Meta-analysis Protocols (INPLASY) database (registration ID: 202410118) and structured by the groundwork by Levac et al. [16] and Arksey and O’Malley [17] and to the checklist by Preferred Reporting Items for Systematic Reviews and Meta-Analyses (PRISMA) Extension for Scoping Reviews [18].

### 2.1 Search strategy

A block search method including three concept blocks, *Appetite, Older Adults, and Physical Activity*, was used. Articles required at least one term from each block, combined with “OR” within blocks and “AND” between blocks. The search, conducted in PubMed, Web of Science, and CINAHL, used MeSH terms and title/abstract keywords. Terms for older adults included: *Aged, Aging, Older, Aged, 80 and over* and *Elderly*; for appetite: *Appetite, Hunger,* and *Satiety;* and for physical activity: *Exercise, Physical Activity, Exercise Therapy, Physical Fitness, Resistance Training* and *Sport.* A librarian with expertise in public health, review methodology, and study designs was consulted to select databases and search terms. Search details are in Supplemental Tables A1 and A2. All references were managed using the bibliographic software Rayyan (Rayyan.ai, Qatar Foundation, State of Qatar) [19] and Zotero [20]. Duplicates were detected using Rayyan and these were manually removed. The database searches were conducted on November 23, 2023, and included all studies available up to that date.

### 2.2 Eligibility criteria

The eligibility criteria for the included studies were as follows: English language; human studies; target population aged over 55 years without severe diseases, specifically cancer, late-stage dementia/Alzheimer’s or Covid-19, as these conditions may affect appetite to a large extent; addressing associations between appetite and physical activity; original articles; not qualitative studies, since our focus was on quantifying associations between appetite and physical activity. Studies involving participants with conditions like type 2 diabetes mellitus, obesity, and frailty were included for a more representative view of appetite regulation in older adults. An age threshold of 55 was chosen to capture a broader sample of older adults and maximize study inclusion. However, 22 of the 25 included studies had a mean age over 65.

### 2.3 Identifying relevant articles

**Fig. 1:**
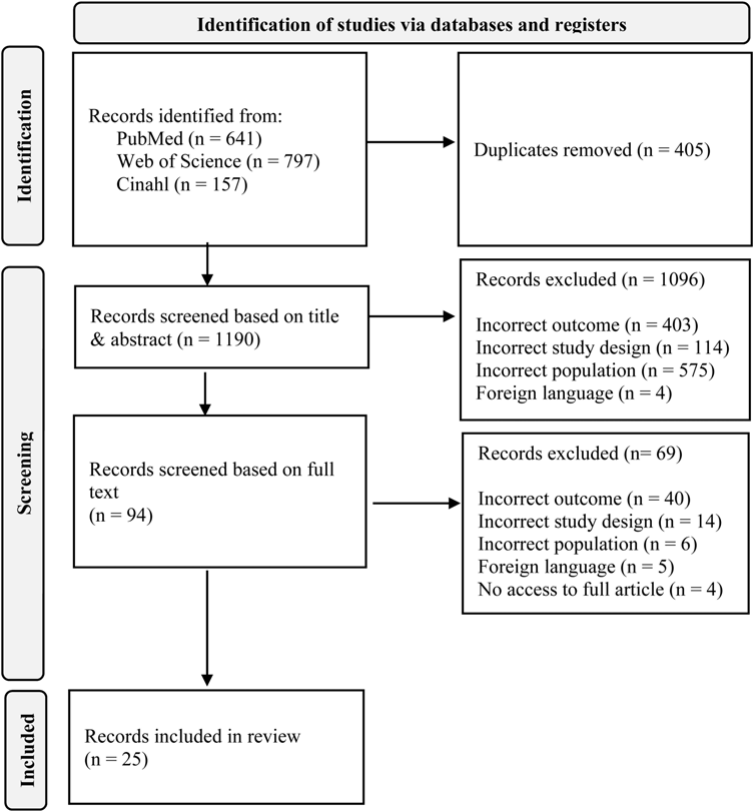
Flow diagram of the screening process.

The first step, consisting of screening titles and abstracts, was performed by two independent readers using the program Rayyan in blind mode [19]. The second step, performed by two readers, consisted of reading the full length of the included articles identified in step one (n = 94). A total of 25 articles were finally included, see the screening process in Fig. 1. Lack of agreement during the screening process was solved by discussions between the authors A.T and A.K for the first step and between A.T and M.N for the second step.

### 2.4 Data extraction

Information about the authors, country, study design, participants, methods used to assess physical activity and appetite, and main findings, were extracted. When charting the data, it was categorized according to study design. Since this was a scoping review, no quality appraisal was conducted for this study.

## 3 Results

Although 25 articles were included (Fig. 1) in this review, a meta-analysis was not conducted due to the heterogeneity in study designs.

### 3.1 Study characteristics

A total of 1190 unique articles were identified and, after screening, 25 were included in this review. Of those included, 14 were cross-sectional studies, seven were randomized controlled trials (RCT), three were non-randomized controlled trials, and one was a longitudinal observational study. The studies were from: USA (n = 4), Netherlands (n = 4), UK (n = 4), Denmark (n = 4), Japan (n = 3), Poland (n = 2), Sweden (n = 2), Germany (n = 1), Scotland (n = 1), Turkey (n = 1), Taiwan (n = 1), Spain (n = 1), Finland (n = 1), Ethiopia (n = 1), and Iran (n = 1). Two were conducted as collaborations between several of the mentioned countries. Participants, without severe diseases, ranged in age from 55 to 95 years. Females made up 52% of the total population and most studies involved community-dwelling older adults, of which three involved persons receiving home care and two involved hospital inpatients (due to conditions such as moderate heart failure, frailty, early dementia, fractures or respiratory illness) or long-term care residents. Information about the study characteristics is presented in Table 1 (observational studies) and Table 2 (non-randomized and randomized trials). Key findings and study limitations are summarized in Table 3.

**Table 1:** Characteristics of the included observational studies.

Reference	Country	Study design	Participants	Appetite assessment	Physical activity assessment	Main findings
Apolzan et al., 2009 [34]	US	Cross-sectional study	32 community-dwelling adults (16 females, 16 males), 68−73 years (mean age 70.5)	Self-reported scale (1−13) on hunger, fullness, and desire to eat.	Paffenberger Physical Activity Questionnaire	No significant associations between physical activity and appetite-related measures
Andreae et al., 2019 [35]	Sweden	18-month longitudinal study	186 outpatients (56 females, 130 males), 60−82 years (mean age 70.7)	Council on Nutrition Appetite Questionnaire (CNAQ) at baseline and 18-month follow-up	Daily steps via accelerometer for 7 days, assessed at baseline and 18-month follow-up.	More daily steps were linked to better self-reported appetite at both time points.
Berggren et al., 2020 [30]	Sweden	Cross-sectional study	121 home care residents (82 females, 39 males), 81−86 years (mean age 83.5)	Functional Assessment of Anorexia/Cachexia Subscale Therapy	Patient-Generated Subjective Global Assessment Short Form	Older adults at risk of malnutrition had worse appetite and lower physical activity.
Cox et al., 2022 [23]	UK	Cross-sectional study	200 hospital inpatients (80 females, 120 males), 74−88 years (mean age 80.7)	Simplified Nutritional Appetite Questionnaire (SNAQ)	Physical Activity Scale for the Elderly	Higher physical activity levels and better mood were linked to improved appetite.
Dermott et al., 2009 [32]	US	Cross-sectional study	93 long-term care residents (59 females, 34 males), 65−90 years (mean age 81)	CNAQ	Self-reported frequency and intensity of weekly physical activities	No association found between physical activity and appetite.
Hung et al., 2019 [27]	Netherlands, UK, Finland, Spain, and Poland	Cross-sectional study (online survey in 5 EU-countries)	1825 community-dwelling adults (905 females, 920 males), 65−74 years	SNAQ	IPAQ	Lower levels of physical activity were associated with poorer appetite.
Janus et al., 2019 [25]	Denmark	Cross-sectional study	1326 community-dwelling adults with overweight (623 females, 703 males), 59−73 years (mean age 66)	Blood sample for fasting GLP-1 concentrations	Combined heart rate monitors and accelerometers, measured over 7 days	Physical activity was associated with lower fasting GLP-1 concentrations in older men with overweight.
Jeruszka-Bielak et al., 2022 [22]	Poland	Cross-sectional study	361 community-dwelling and home care adults (313 females, 48 males), 60−89 years (mean age 69.5)	SNAQ	Minnesota Leisure Time Activity Questionnaire	Higher physical activity levels were linked to improved appetite and higher food intake.
Kimura et al., 2018 [33]	Japan	Cross-sectional study	205 outpatients with early-stage Alzheimer’s Disease (score of 21 or higher in the Mini-Mental State Examination) (130 females, 75 males), 65−89 years (mean age 77.2)	CNAQ	Self-reported questions on moderate- and low-intensity levels of physical activity	Poor appetite, but not low physical activity level, was associated with sarcopenia.
van der Pols-Vijlbrief et al., 2016 [26]	Netherlands	Cross-sectional study	300 home care adults (205 females, 95 males), 65 or older (mean age 81.7)	SNAQ	Self-reported number of days/week that moderate physical activity was performed for ≥30 min	Physical inactivity and poor appetite were associated with increased risk of undernutrition.
Tsai et al., 2023 [21]	Netherlands, US, Denmark	Cross-sectional study	1173 community-dwelling adults (745 females, 428 males), 53−80 years	Self-reported appetite as good, not always good, or not good	Hip and wrist accelerometers for 7 days	Higher physical activity (hip accelerometer) improved appetite and increased sedentary behaviour, decreased appetite.
Tsai et al., 2006 [28]	Taiwan	Cross-sectional study	4440 community-dwelling adults (2080 females, 2360 males), 53−80 years	Self-reported appetite as good, not always good or not good	In-home questionnaire interview	Decreased appetite was associated with reduced food intake, and increased physical activity was associated with increased food intake.
Tsutsumimoto et al., 2017 [31]	Japan	Cross-sectional study	4417 community-dwelling adults (2328 females, 2089 males), 72−80 years (mean age 75.8)	SNAQ	Self-reported questions on moderate- and low-intensity levels of physical activity	Anorexia of aging/appetite loss was not associated with low levels of physical activity but with slowness, exhaustion, and weight loss.
Uymaz et al., 2021 [24]	Turkey	Cross-sectional study	669 community-dwelling adults (367 females, 302 males), 65 or older (mean age 70.9)	Power of Food Scale	Self-reported exercise frequency questions (never, 1−2 days/week, 3+ days/week)	Hedonic hunger increased 0.51 times in those doing sports 3+ days/week vs. no physical activity.
Zewdu et al., 2023 [29]	Ethiopia	Cross-sectional study	634 community-dwelling adults (341 females, 293 males), 65 years and above (mean age 74.6)	Self-reported appetite as normal, good, or poor appetite	IPAQ-Short Form	Poor appetite and low physical activity were linked to increased risk for malnutrition.

**Table 2:** Characteristics of the included non-randomized and randomized controlled trials.

Reference	Country	Study design	Participants	Appetite assessment	Physical activity assessment	Main findings
**Non-randomized controlled trials**						
Ahmadi et al., 2018 [37]	Iran	8-week quasi-experimental trial	30 community-dwelling males, 60−70 years (mean age 69.1)	Ghrelin levels via blood samples, pre-and post-intervention	4-weekly 60-min aerobic sessions, measured via heart rate sensor, control group inactive	Small increase in ghrelin levels in the exercise group
Muller et al., 2017 [38]	Denmark	3-day non-randomized trial	13 community-dwelling adults with type 2 diabetes (5 females, 8 males), 63−67 years (mean age 65)	Self-reported hunger, fullness and food consumption; blood samples for ghrelin, leptin and PYY, assessed pre- and post-intervention	2 × 60-min supervised interval walking sessions (control group: continuous walking)	Interval walking increased fullness and reduced hunger shortly after the session compared to low-intensity walking.
Rosenkilde et al., 2015 [36]	Denmark	2-week non-randomized trial	6 community-dwelling males, 57−65 years (mean age 61.3)	Hunger, satiety, fullness, and food intake via VAS twice/day; blood samples for glucose, insulin, leptin, ghrelin, PYY and GLP-1, assessed pre- and post-intervention	14 days of cycling with heart monitor	Exercise increased morning and evening hunger; concentrations of insulin, GLP-1 and PYY increased post-intervention
**Randomized controlled trials**						
Apolzan et al., 2011 [49]	US	5-day randomized controlled trial	34 community-dwelling adults (18 females, 16 males), 60−70 years (mean age 75)	Self-reported scale (1−13) on hunger, fullness and desire to eat; blood sampling for glucose, insulin, ghrelin, cholecystokinin (CCK), glucagon-like peptide-1(GLP-1), measured multiple times daily	Yale Physical Activity Questionnaire, activity monitors for 3 days, maximum strength test. Grouped as resistance trained (resistance training ≥2 times/week during the previous 6 months) vs. sedentary	No differences in postprandial appetite, metabolic or endocrine responses between the groups
De Jong et al., 2000 [42]	Netherlands	17-week randomized controlled trial	159 community-dwelling adults (113 females, 46 males), 72−86 years (mean age 78.7)	29-questions on appetite, hunger, taste and smell perception, assessed pre- and post-intervention	2-weekly 45-min group training sessions led by a trainer and supervised by a project leader	No effect of exercise intervention on appetite
Diekmann et al., 2019 [41]	Germany	4-day randomized controlled trial (each day separated by 2 weeks)	26 adults with overweight (8 females, 18 males), 60−80 years (mean age 69.9)	Visual analogue scale (VAS) for hunger, appetite and satiety, assessed at baseline and intervals up to 4.5 h post-meal	30-min supervised walking session post-meal, measured with pedometers	No effect of physical activity on appetite or hunger
Diekmann et al., 2019 [41]	Germany	4-day randomized controlled trial (each day separated by 2 weeks)	26 adults with overweight (8 females, 18 males), 60−80 years (mean age 69.9)	Visual analogue scale (VAS) for hunger, appetite and satiety, assessed at baseline and intervals up to 4.5 h post-meal	30-min supervised walking session post-meal, measured with pedometers	No effect of physical activity on appetite or hunger
Diekmann et al., 2019 [41]	Germany	4-day randomized controlled trial (each day separated by 2 weeks)	26 adults with overweight (8 females, 18 males), 60−80 years (mean age 69.9)	Visual analogue scale (VAS) for hunger, appetite and satiety, assessed at baseline and intervals up to 4.5 h post-meal	30-min supervised walking session post-meal, measured with pedometers	No effect of physical activity on appetite or hunger
Johnson et al., 2021 [40]	UK	12-week randomized controlled trial	39 community-dwelling adults, 61−71 years (mean age 66)	Hunger, satiety, fullness, and prospective food consumption via VAS; blood samples for insulin and leptin, assessed at baseline, week 6, and week 12	2 supervised resistance training sessions per week	Appetite response increased after the 12-week intervention, but not after 6 weeks.
Jonson et al., 2021 [39]	UK	2-day randomized controlled trial	20 community-dwelling adults (13 females, 7 males), 63−73 years (mean age 68)	Hunger, satiety, fullness, and prospective food consumption via VAS, assessed pre-intervention and 2 h post-intervention	2 × 1-h supervised resistance exercise sessions	Resistance exercise session temporarily reduced appetite response 30 min after the session.
Kimura et al., 2013 [43]	Japan	12-week cluster randomized controlled trial	92 community-dwelling adults (75 females, 17 males), 65−90 years (mean age 74.3)	CNAQ	1-h exercise biweekly and recommendations of 10 min of physical activity 2−3 times/day at home	The exercise intervention did not affect the appetite response.
Ofosu et al., 2023 [44]	Scotland	12-week randomized controlled trial	49 home care adults (42 females, 7 males), 65 or older	SNAQ, assessed pre- and post-intervention	3 supervised movement sessions per week	No improvement in appetite, but physical activity improved anxiety, depression, and sleep.

**Table 3:** Summary of included studies: Study Design, Key Findings and Limitations.

Reference	Study Design	Key Findings	Limitations
Ahmadi et al., 2018 [37]	Non-randomised trial (8 weeks)	Aerobic training appeared to enhance ghrelin gene expression as well as reduced BMI and body fat percentage in older men.	A small sample size (n = 30) and only males, subjects were selected by convenience sampling.
Andreae et al., 2019 [35]	Observational longitudinal study (18 months)	More daily steps were linked to better self-reported appetite, and those who were more physically active had better appetite.	High dropout rate and findings are limited to outpatients with moderate heart failure.
Apolzan et al., 2009 [34]	Cross-sectional study	No significant associations between physical activity and appetite-related measures.	No power calculation was conducted to determine the sample size, and appetite and mood analyses were included as secondary objectives in a study examining the effects of physical activity and age on inflammation.
Apolzan et al., 2011 [49]	Randomized controlled trial (5 days)	No differences in postprandial appetite, metabolic or endocrine responses between the groups.	Usage of an equal interval scale instead of a labelled magnitude scale as appetite ratings were low for the fasted state, as well as appetite was assessed between groups for resistance training, and it’s unclear if participants responded similarly to different intensities.
Berggren et al., 2020 [30]	Cross-sectional study	Older adults at risk of malnutrition had worse appetite and lower physical activity.	A small sample size that limits generalizability, some patients declined to participate due to tiredness or serious illness, possibly excluding the frailest and most at-risk individuals. The study was also limited by its reliance on questionnaire data.
Cox et al., 2022 [23]	Cross-sectional study	Higher physical activity levels and better mood were linked to improved appetite.	Included hospitalized older adults with relatively high function and cognition, possibly underestimating poor appetite prevalence. Physical activity data covered only the 7 days before hospitalization, potentially reflecting reduced activity due to acute illness.
De Jong et al., 2000 [42]	Randomized controlled trial (17 weeks)	No effect of exercise intervention on appetite.	A longer-term study would be needed to determine effects on outcomes like appetite.
Dermott et al., 2009 [32]	Cross-sectional study	No association found between physical activity and appetite.	The cross-sectional design and low number of participants were physically active.
Diekmann et al., 2019 [41]	Randomized controlled trial (4 days, each day separated by 2 weeks)	No effect of physical activity on appetite or hunger.	This is an explorative analysis of secondary outcome measures and the selected time points for measurements of appetite may not fully represent the postprandial period.
Hung et al., 2019 [27]	Cross-sectional study	Lower levels of physical activity were associated with poorer appetite.	Results are only valid for older adults without cognitive impairment, data were collected online, limiting the sample to those with internet access and basic internet skills, and the study relied on self-reported data, which may affect accuracy.
Janus et al., 2019 [25]	Cross-sectional study	Physical activity was associated with lower fasting GLP-1 concentrations in older men with overweight.	Cross-sectional design and men and women were not matched, which could have been beneficial as there were significant differences between men and women in time spent on moderate-intensity physical activity.
Jeruszka-Bielak et al., 2022 [22]	Cross-sectional study	Higher physical activity levels were linked to improved appetite and higher food intake.	Small sample size, cross-sectional design, indexes were based on self-reported data.
Johnson et al., 2021 [40]	Randomized controlled trial (12 weeks)	Appetite response increased after the 12-week intervention, but not after 6 weeks.	Supervised resistance training may not reflect real-life scenarios, participants were active, healthy older adults, possibly not representative of those with comorbidities affecting appetite, and total energy expenditure, and habitual physical activity were not measured.
Johnson et al., 2021 [39]	Randomized controlled trial (2 days)	Resistance exercise session temporarily reduced appetite response 30 min after the session.	Participants were healthy, active older adults, so findings may not apply to frail individuals, no mechanistic measures (e.g., appetite-related hormones) were taken, and the study assessed only acute appetite response.
Kimura et al., 2013 [43]	Randomized controlled trial (12 weeks)	The exercise intervention did not affect the appetite response.	The study was not an ideal randomized controlled trial, as participants were grouped based on their home address to reduce transportation barriers, the sample size was small, and predominantly female participants.
Kimura et al., 2018 [33]	Cross-sectional study	Poor appetite, but not low physical activity level, was associated with sarcopenia.	Cross-sectional design and reliance on self-reported physical activity.
Muller et al., 2017 [38]	Non-randomized trial (3 days)	Interval walking increased fullness and reduced hunger shortly after the session compared to low-intensity walking.	Small sample size (n = 13) and assessing only the acute effect of training on appetite.
Ofosu et al., 2023 [44]	12-week randomized controlled trial	No improvement in appetite, but physical activity improved anxiety, depression, and sleep.	Small sample size (n = 49) and a high dropout rate.
van der Pols-Vijlbrief et al., 2016 [26]	Cross-sectional study	Physical inactivity and poor appetite were associated with increased risk of undernutrition.	Small sample size and reliance on self-reported physical activity.
Rosenkilde et al., 2015 [36]	2-week non-randomized trial	Exercise increased morning and evening hunger; concentrations of insulin, GLP-1 and PYY increased post-intervention.	Non-random subject selection and small sample size (n = 6).
Tsai et al., 2006 [28]	Cross-sectional study	Decreased appetite was associated with reduced food intake, and increased physical activity was associated with increased food intake.	Cross-sectional design and self-reported physical activity.
Tsai et al., 2023 [21]	Cross-sectional study	Higher physical activity (hip accelerometer) improved appetite and increased sedentary behaviour, decreased appetite.	Cross-sectional design and self-reported physical activity including categories that may not directly correspond to moderate and vigorous physical activity levels.
Tsutsumimoto et al., 2017 [31]	Cross-sectional study	Anorexia of aging/appetite loss was not associated with low levels of physical activity but with slowness, exhaustion, and weight loss.	Cross-sectional design and non-random recruitment of relatively healthy older adults.
Uymaz et al., 2021 [24]	Cross-sectional study	Hedonic hunger increased 0.51 times in those doing sports 3+ days/week vs. no physical activity.	Cross-sectional design and chronic diseases and drug use among the participants were not considered.
Zewdu et al., 2023 [29]	Cross-sectional study	Poor appetite and low physical activity were linked to increased risk for malnutrition.	Cross-sectional design and reliance on self-reported physical activity.

### 3.2 Appetite assessment

The most prevalent methods for assessing appetite in the included studies were questionnaires (e.g., the Simplified Nutritional Appetite Questionnaire (SNAQ)) and the Visual Analogue Scale (VAS)). Four studies assessed appetite using questionnaires and measurements of appetite-regulating hormones in the blood, such as ghrelin, Glucagon-like Peptide-1 (GLP-1), Peptide YY (PYY), Cholecystokinin (CCK), leptin, and insulin. Two studies assessed appetite solely through hormone measurements.

### 3.3 Physical activity assessment

It is important to distinguish between physical activity and exercise, since the usage of these terms varied across the studies. Physical activity includes all types of bodily movement, while exercise is a subset of physical activity that is intentional, structured, and planned to improve or maintain physical fitness. Furthermore, the type of physical activity and/or exercise in the included studies varied between resistance training, aerobic training, habitual physical activity, and multicomponent physical activity (i.e., resistance training, aerobic training, and habitual physical activity), where multicomponent physical activity was most prevalent. The most common methods used to assess physical activity were self-reported questionnaires and accelerometers.

### 3.4 Categorization based on study design

The studies are categorized by the following study designs: cross-sectional studies, longitudinal observational studies, non-randomized trials, and RCTs. One RCT focused on a food intake intervention rather than an exercise intervention, comparing resistance-trained and sedentary participants. Different types of food were provided and appetite was assessed using a self-reported scale along with measurements of insulin, glucose, ghrelin, CCK and GLP-1. Appetite responses were similar between the groups, except for higher postprandial levels of CCK, a satiety-signaling hormone, in the resistance-trained group.

### 3.5 Cross-sectional studies

This category includes 14 cross-sectional studies investigating the relationship between physical activity and appetite in older adults. Three studies [[21], [22], [23]] found that higher levels of physical activity were associated with better self-reported appetite using the SNAQ, measured as “good”, “not always good”, “not good”. Additionally, engaging in sports three or more times a week was linked to increased hedonic hunger [24]. Another study involving 1326 older adults showed that higher levels of physical activity, measured using accelerometers, were associated with lower fasting GLP-1 levels (a satiety hormone, potentially indicating increased appetite) in men, but not women [25].

Five studies [[26], [27], [28], [29], [30]] found that poor appetite was associated with lower levels of physical activity, while four studies [[31], [32], [33], [34]] found no significant associations. Methods used to assess appetite varied, and included the SNAQ, the Council on Nutrition Appetite Questionnaire (CNAQ), and scales addressing hunger and desire to eat. A common factor among the four studies that found no significant associations was their usage of self-reported data to assess physical activity levels.

### 3.6 Longitudinal observational studies

One 18-month longitudinal observational study (n = 186) [35] was included in this review. This study found an association between higher levels of physical activity (assessed using both questionnaire and accelerometer) and improved appetite assessed using CNAQ.

### 3.7 Non-randomized controlled trials

Three non-randomized controlled studies were included in this category. In a small non-randomized trial (n = 6) on high-intensity exercise, participants cycled for 14 days and reported their subjective appetite twice daily; they also measured levels of glucose, insulin, leptin, ghrelin, and citrulline pre-and post-intervention. Results showed increased hunger in the evening and morning and increased levels of fasting plasma concentrations of insulin, GLP-1 and PYY [36]. Similarly, another high-intensity intervention with 30 participants performing four weekly aerobic sessions for eight weeks showed a small increase in plasma ghrelin, an appetite-regulating hormone that signals hunger, post intervention [37].

In contrast, another non-randomized trial [38] on low-intensity exercise (walking sessions on a treadmill) in 13 older adults with type 2 diabetes found no changes in appetite-regulating hormones; however, there was an increase in self-reported fullness directly after the intervention.

### 3.8 Randomized controlled trials

Of the included RCTs, two found an association between resistance training and appetite. One of these [39] assessed appetite using VAS after single exercise sessions in 20 older adults and found a temporarily reduced appetite response directly after exercise. Another RCT [40] including 39 participants investigated the effect of resistance training for 12 weeks on appetite assessed using both VAS and measurements of leptin, ghrelin, PP, and PYY levels in the blood. The study found significant increases in self-reported appetite after intervention and a decrease in leptin levels, however, there was no effect on ghrelin, PP or PYY.

Four RCTs [[41], [42], [43], [44]] examined the association of a physical activity intervention on appetite by assessing appetite using only subjective methods (i.e., VAS, SNAQ, CNAQ, Likert Scale) and found no associations. The number of participants ranged between 26 and 159. In all the studies, physical activity was of low intensity, for example walking, or low frequency exercise, such as one hour of exercise every two weeks.

## 4 Discussion

This scoping review of 25 studies found associations between physical activity and appetite in all non-randomized studies, 11 of 15 observational studies, and two of seven randomized trials. Higher-intensity exercise showed stronger links to appetite, while four cross-sectional studies and five RCTs found no associations. Research is limited by a lack of long-term RCTs and varied methodologies, with some studies examining acute appetite response and others focusing on long-term effects.

As in the majority of the included studies showing associations between physical activity and appetite, a meta-analysis by Hubner et al. [11] found similar results among older adults. However, unlike the present study, which focuses solely on appetite, this meta-analysis examined both appetite and energy intake, and revealed that physical activity can influence resting hunger and satiety, promote appetite control, and lead to a balanced energy intake. In contrast, another systematic review [12] found insufficient evidence to support a relationship between physical activity, appetite, and energy intake in older adults, however, both reviews emphasize other benefits of physical activity.

The benefits of physical activity and exercise are numerous. In addition to maintaining muscle mass and strength, it may also increase appetite and energy intake through social factors, especially when exercising with others. Loneliness is associated with lower food intake among older adults [45] and group exercise can help reduce these feelings of loneliness. For example, Cox et al. [23] found positive associations between physical activity, mood, and appetite. Similarly, Ofosu et al. [44] reported no effect of physical activity on appetite, although improvements in anxiety and depression were observed, highlighting other benefits. The mechanisms behind this relationship are not established; however, evidence shows that exercise releases hormones that improve mood and reduce anxiety, stress, and depression [46].

One study included by Berggren et al. [30] found no association between physical activity and appetite; however, they found that sarcopenia was associated with poorer appetite. Since sarcopenia leads to reduced muscle mass, a potential mechanism for this association is that muscle, being a metabolically active tissue, requires energy homeostasis alongside energy expenditure, and therefore the brain may signal increased hunger and appetite. This is also discussed by Grannell et al. [47] who noted a positive correlation between fat-free mass, the main component of muscle, and energy intake. Additionally, de Jong et al. [42] reported no effect of physical activity on appetite but observed improvements in energy intake and muscle mass. This highlights the importance of resistance training and underscores the differing outcomes of physical activity and exercise. Unlike general physical activity, resistance training is purposeful, aiming to increase muscle strength and mass, which could potentially lead to higher energy intake.

Interestingly, some studies reported a link between physical activity and increased appetite without a corresponding change in energy intake [39], while others found no appetite change but improved energy intake with physical activity [42,48]. This emphasizes appetite loss and reduced food intake as different phenotypes. However, these observed differences in appetite and energy intake may also be due to variation in study design and methodology. For instance, subjective methods for assessing appetite ranged from questionnaires to scales, while objective methods involved measuring appetite-regulating hormones in the blood. This variability complicates direct comparisons, since different hormones may play distinct roles in appetite regulation. Additionally, the terminology used to describe appetite varies across studies, with some addressing hunger and satiety, and others focusing on hedonic hunger, which drives eating for pleasure without an energy deficit [24]. Appetite regulation is complex, including sensations like hunger, satiety, and fullness, making consistent measurements difficult. Conditions such as the anorexia of aging or malnutrition are sometimes confused with appetite loss, despite not being the same thing.

The studies included vary concerning when appetite was assessed, with some measuring acute responses immediately after exercise and others evaluating long-term responses after weeks of regular exercise. While many findings indicate a positive relationship between appetite and physical activity, Apolzan et al. [49] observed increased CCK levels, which signals satiety, reduced hunger and desire to eat immediately after resistance training. This highlights the variability in timing in the studies and its impact on appetite response. For instance, one study [40] observed improved appetite after 12 weeks of resistance training but not after six weeks, suggesting long-term training may be necessary to influence appetite. In contrast, a systematic review [50] provided more evidence for an appetite-reducing effect of long-term physical activity rather than an appetite-increasing effect.

Continuing with the topic of mechanisms behind this relationship, it has been suggested that gastric emptying is accelerated in those who are physically active, leading more quickly to an empty stomach and, as a result, a feeling of hunger or increased appetite [6]. The hormone ghrelin stimulates gastric emptying [45] and, as observed in one of the included studies by Ahmadi et al. [37], plasma ghrelin levels increased after an exercise intervention leading to increased hunger and appetite, which supports the hypothesis that physical activity has the potential to increase appetite.

The included studies varied in their focus on physical activity and exercise, which are related but distinct concepts. In frail older adults, these distinctions are important due to physical limitations and varying physiological responses. Higher-intensity exercise was often linked to appetite increases, whereas lower-intensity physical activity showed no clear association. For example, Rosenkilde et al. [36] observed improved appetite after 14 days of cycling, likely due to its structured, high intensity nature, which can stimulate energy balance mechanisms and promote hunger [51,52]. In contrast, resistance training, another form of exercise, may enhance appetite over time by promoting muscle mass growth and increasing protein needs, thereby stimulating hunger and overall appetite [11]. This highlights the potential long-term metabolic adaptations induced by exercise. However, high intensity exercise may be impractical for frail older adults due to physical limitations and safety concerns. Lower-intensity physical activity, such as walking, remains more accessible but may not have the same impact on appetite regulation as structured exercise. Additionally, responses to activity can differ depending on factors such as age, baseline activity level, and health status. For instance, a relatively active 60-year-old may exhibit different appetite responses and have greater capacity to engage in exercise compared to a frail 90-year-old, further emphasizing the need to distinguish between general movement and structured training when assessing appetite effects in this specific group.

Another factor that should be taken into account when analyzing the results is the country in which the studies have been conducted, with the majority of studies being from high-income countries (HICs), such as the USA, UK, and northern Europe. Findings from HICs may not be generalizable to populations in low-income countries (LICs) due to differences in lifestyle, social and cultural norms, genetics, environment, and socioeconomic factors [53]. For example, malnutrition is more prevalent in LICs compared to HICs, and while research primarily conducted in HICs shows a correlation between malnutrition and appetite loss, it remains unclear if this relationship holds in LICs [54].

### 4.1 Strengths and limitations

One strength of this scoping review is its extensive mapping of studies on this topic, providing a summary of existing knowledge and highlighting gaps for future research. A scoping review allows more flexibility, which facilitates a broader range of methodologies and perspectives. However, this broad approach can make it difficult to draw clear conclusions or recommendations, although this is not the primary aim of a scoping review. Another limitation is that many studies relied on self-reported measures of appetite and physical activity, which can produce unreliable data, particularly in older adults with mild cognitive impairment. Additionally, we acknowledge that qualitative studies were not included, which could have provided valuable insights, however, our focus was to examine the associations between appetite and physical activity using quantitative data.

### 4.2 Existing research gaps

Future studies should investigate the sequence of events regarding physical activity and changes in appetite in older adults [21]. Additionally, examining the biological mechanisms underlying the relationship between physical activity and appetite is important for understanding causality [25,26,35]. Lastly, there is a need for long-term randomized interventions that investigate the association between exercise and appetite [32].

## 5 Conclusion

The majority of the included studies reported a link between physical activity and appetite in older adults, but methodological variations hinder direct comparisons. Research on frail older adults is limited, with studies differing in short- and long-term focus. Future research should explore exercise duration, intensity, and social engagement in appetite regulation. More RCTs with consistent methods are needed to clarify these relationships and to develop effective exercise strategies for older adults.

## CRediT authorship contribution statement

Methodology and validation: A.T., A.K., and M.N. Conceptualization, formal analysis and writing original draft: A.T. Writing, reviewing and editing: A.T., A.K., M.N., and P.S.B.

## Funding

10.13039/501100004359Swedish Research Council (2022-06295).

## Declaration of competing interest

The authors have no conflicts of interest to declare.
