# Supplementary material for: The relationship between physical activity and appetite among older adults — A scoping review
Source: J Nutr Health Aging. 2025 Mar 23;29(5):100538. doi: 10.1016/j.jnha.2025.100538 (PMC12180069; doi:10.1016/j.jnha.2025.100538)
Supplement: Supplementary file 1 [file mmc1.docx]

**Supplemental Material**

**List of included material**

1. Table A1. Databases and search terms
2. Table A2. Documentation of the search in PubMed

| Table A1. Databases and search terms. | | |
| --- | --- | --- |
| Databases: PubMed, Web of Science, Cinahl | | |
| Block 1: elderly | **Block 2: appetite** | **Block 3: physical activity** |
| Aged (mesh) | Appetite (mesh) | Exercise (mesh) |
| Aging (mesh) | Appetite (ti/ab) | Exercise therapy (mesh) |
| Aged, 80 and over (mesh) | Hunger (ti/ab) | Physical fitness (mesh) |
| Aged (ti/ab) | Satiety (ti/ab) | Exercise (ti/ab) |
| Older (ti/ab) |  | Physical activity (ti/ab) |
| Elderly (ti/ab) |  | Resistance training (ti/ab) |
|  |  | Sport (ti/ab) |

| Table A2. Documentation of the search in PubMed (example). | | | |
| --- | --- | --- | --- |
| Search number | **Search terms** | **Field** | **Number of hits** |
| 1 | Aged OR Aging OR Aged, 80 and over OR Geriatrics | Mesh | [3,679,935](https://pubmed.ncbi.nlm.nih.gov/?term=%28%28%28Aged%5BMeSH+Terms%5D%29+OR+%28Aged%2C+80+and+over%5BMeSH+Terms%5D%29%29+OR+%28Geriatrics%5BMeSH+Terms%5D%29%29+OR+%28aging%5BMeSH+Terms%5D%29&ac=no&sort=relevance) |
| 2 | Aged OR older OR elderly OR geriatric* | Ti/ab | [1,413,902](https://pubmed.ncbi.nlm.nih.gov/?term=Aged%5BTitle%2FAbstract%5D+OR+older%5BTitle%2FAbstract%5D+OR+elderly%5BTitle%2FAbstract%5D+OR+geriatric%2A%5BTitle%2FAbstract%5D&sort=fauth&sort_order=asc&ac=no) |
| 3 | 1 OR 2 |  | [4,428,604](https://pubmed.ncbi.nlm.nih.gov/?term=%28%28%28%28Aged%5BMeSH+Terms%5D%29+OR+%28Aged%2C+80+and+over%5BMeSH+Terms%5D%29%29+OR+%28Geriatrics%5BMeSH+Terms%5D%29%29+OR+%28aging%5BMeSH+Terms%5D%29%29+OR+%28Aged%5BTitle%2FAbstract%5D+OR+older%5BTitle%2FAbstract%5D+OR+elderly%5BTitle%2FAbstract%5D+OR+geriatric%2A%5BTitle%2FAbstract%5D%29&ac=no&sort=relevance) |
| 4 | Appetite | Mesh | [11,708](https://pubmed.ncbi.nlm.nih.gov/?term=Appetite%5BMeSH+Terms%5D&sort=fauth&sort_order=asc&ac=no) |
| 5 | Appetite OR hunger OR satiety | Ti/ab | [49,910](https://pubmed.ncbi.nlm.nih.gov/?term=Appetite%5BTitle%2FAbstract%5D+OR+hunger%5BTitle%2FAbstract%5D+OR+satiety%5BTitle%2FAbstract%5D&sort=fauth&sort_order=asc&ac=no) |
| 6 | 4 OR 5 |  | [53,784](https://pubmed.ncbi.nlm.nih.gov/?term=%28Appetite%5BMeSH+Terms%5D%29+OR+%28Appetite%5BTitle%2FAbstract%5D+OR+hunger%5BTitle%2FAbstract%5D+OR+satiety%5BTitle%2FAbstract%5D%29&sort=fauth&sort_order=asc&ac=no) |
| 7 | Exercise OR Exercise Therapy OR Physical Fitness | Mesh | [308,289](https://pubmed.ncbi.nlm.nih.gov/?term=%28%28Exercise%5BMeSH+Terms%5D%29+OR+%28Exercise+Therapy%5BMeSH+Terms%5D%29%29+OR+%28Physical+Fitness%5BMeSH+Terms%5D%29&sort=fauth&sort_order=asc&ac=no) |
| 8 | Exercise OR physical activity OR resistance training OR sport | Ti/ab | [768,968](https://pubmed.ncbi.nlm.nih.gov/?term=%28Exercise%5BTitle%2FAbstract%5D+OR+physical+activity%5BTitle%2FAbstract%5D%29+OR+%28%28resistance+training%5BTitle%2FAbstract%5D%29+OR+%28sport%29%29&ac=no&sort=relevance) |
| 9 | 7 OR 8 |  | [804,423](https://pubmed.ncbi.nlm.nih.gov/?term=%28%28%28Exercise%5BMeSH+Terms%5D%29+OR+%28Exercise+Therapy%5BMeSH+Terms%5D%29%29+OR+%28Physical+Fitness%5BMeSH+Terms%5D%29%29+OR+%28%28Exercise%5BTitle%2FAbstract%5D+OR+physical+activity%5BTitle%2FAbstract%5D%29+OR+%28%28resistance+training%5BTitle%2FAbstract%5D%29+OR+%28sport%29%29%29&ac=no&sort=relevance) |
| 10 | 3 AND 6 AND 9 |  | [641](https://pubmed.ncbi.nlm.nih.gov/?term=%28%28%28%28%28%28Aged%5BMeSH+Terms%5D%29+OR+%28Aged%2C+80+and+over%5BMeSH+Terms%5D%29%29+OR+%28Geriatrics%5BMeSH+Terms%5D%29%29+OR+%28aging%5BMeSH+Terms%5D%29%29+OR+%28Aged%5BTitle%2FAbstract%5D+OR+older%5BTitle%2FAbstract%5D+OR+elderly%5BTitle%2FAbstract%5D+OR+geriatric%2A%5BTitle%2FAbstract%5D%29%29+AND+%28%28Appetite%5BMeSH+Terms%5D%29+OR+%28Appetite%5BTitle%2FAbstract%5D+OR+hunger%5BTitle%2FAbstract%5D+OR+satiety%5BTitle%2FAbstract%5D%29%29%29+AND+%28%28%28%28Exercise%5BMeSH+Terms%5D%29+OR+%28Exercise+Therapy%5BMeSH+Terms%5D%29%29+OR+%28Physical+Fitness%5BMeSH+Terms%5D%29%29+OR+%28%28Exercise%5BTitle%2FAbstract%5D+OR+physical+activity%5BTitle%2FAbstract%5D%29+OR+%28%28resistance+training%5BTitle%2FAbstract%5D%29+OR+%28sport%29%29%29%29&ac=no&sort=relevance) |
